# Publisher Correction: The persistence of memory in ionic conduction probed by nonlinear optics

**DOI:** 10.1038/s41586-024-07124-6

**Published:** 2024-01-30

**Authors:** Andrey D. Poletayev, Matthias C. Hoffmann, James A. Dawson, Samuel W. Teitelbaum, Mariano Trigo, M. Saiful Islam, Aaron M. Lindenberg

**Affiliations:** 1 Stanford Institute for Materials and Energy Sciences, SLAC National Laboratory, Menlo Park, CA USA; 2 Department of Materials Science and Engineering, Stanford University, Stanford, CA USA; 3 Department of Materials, University of Oxford, Oxford, UK; 4 Linac Coherent Light Source, SLAC National Accelerator Laboratory, Menlo Park, CA USA; 5 Chemistry, School of Natural and Environmental Sciences, Newcastle University, Newcastle upon Tyne, UK; 6 Centre for Energy, Newcastle University, Newcastle upon Tyne, UK; 7 Stanford PULSE Institute, SLAC National Accelerator Laboratory, Menlo Park, CA USA; 8 Department of Chemistry, University of Bath, Bath, UK; 9 Present Address: Department of Physics, Arizona State University, Tempe, AZ USA

**Keywords:** Terahertz optics, Atomistic models, Statistical physics, Batteries, Batteries

Correction to: *Nature* 10.1038/s41586-023-06827-6 Published online 24 January 2024

In the version of the article initially published, there was an error in Fig. 3a where the shaded regions appeared as solid colour. This has now been corrected in the HTML and PDF versions of the article.

