# Supplementary material for: The persistence of memory in ionic conduction probed by nonlinear optics
Source: Nature. 2024 Jan 24;625(7996):691–6. doi: 10.1038/s41586-023-06827-6 (PMC10808053; doi:10.1038/s41586-023-06827-6)
Supplement: Supplementary file 1 — Supplementary Notes 1–6, including Supplementary Figs 1–12 and Supplementary Table 1. [file 41586_2023_6827_MOESM1_ESM.pdf]

nature portfolio

https://doi.org/10.1038/s41586-023-06827-6

Supplementary information

The persistence of memory in ionic conduction probed by nonlinear optics

In the format provided by the authors and unedited

The Persistence of Memory in Ionic Conduction Probed by Nonlinear Optics: Supplementary Information

Supplementary Note 1: Terahertz Kerr Effect 1
Supplementary Note 2: TKE Control Measurements 2
Supplementary Note 3: Optical Conductivity from THz Transmission 6
Supplementary Note 4: Terahertz Pumps in Molecular Dynamics 7
Supplementary Note 5: Logical and Thermodynamic Reversibility 9
Supplementary Note 6: Attempt Frequencies in Na *β*-alumina 11

Figures S1-S12
Table S1
Supplementary References 1-76

Supplementary Note 1: Terahertz Kerr Effect

The 2D terahertz Kerr effect measurement is a $\chi^{(3)}$ measurement that relies on the third-order response function[1,2]:

$$R^{(3)}_{ijkl}(t'',t') = \left(\frac{i}{\hbar}\right)^2 tr\langle \alpha_{ij}(t''+t')\left[\mu_k(t')[\mu_l(0),\rho]\right]\rangle \quad \text{(S1)}$$

Here, the transition dipole moment operator μ and polarizability operator α act on the equilibrium density matrix ρ describing the thermal population of vibrational states. The pump pulses arrive at times *0* and *t'*. The 800nm probe pulse at *t''+t'* is assumed to be short. Because we use one pump pulse, our experiment represents a line cut through the (*t'*, *t''*) space corresponding to *t'=0*. The third-order polarization in the sample is given by the integral over pathways:

$$P_i(t) = \int\int dt'dt'' R^{(3)}_{ijkl}(t'',t') E_{probe,j}(t) E_{THz,k}(t-t'') E_{THz,l}(t-t'-t'') \quad \text{(S2)}$$

This formalism includes all possible excitation pathways via either bound vibrational states, virtual states (Raman), or "thermal bath" states[1,2]. The generated field is the time derivative of the overall ionic and electronic polarization[3–5]:

$$E_{probe}(z,t) - E(z,t) \propto \frac{\partial}{\partial t}\Big(P(z,t+t'')E_{probe}(z,t)\Big) \quad \text{(S3)}$$

Models of coherently excited vibrational responses utilize an oscillatory form of the generated polarization corresponding to a bound vibrational state, including possible anharmonic potential energy surfaces[5,6], anharmonic couplings to other vibrations[1,7], or couplings to a thermal bath[8,9]. For such an oscillating polarization, the detected change in the probe field at any time is proportional to the velocity of the oscillator. However, this formalism does not impose a coherence requirement or a functional form on the trajectory of the charged species interacting with the pump and probe fields. In principle, this treatment should be valid for Debye relaxation[9] or an ensemble of coupled double-well oscillators coupled to a thermal bath and stochastically switching their positions. Here, the ionic polarization *P* arises from coherent displacements of vibrational modes or bound dipoles Q_i, plus an additional incoherent component representing hopping rates *H*. The detected third-order signal is proportional to the transient anisotropy of the hopping rate, measured as a function of the pump-probe time delay. The pump-probe time delay is *t''* in the above formalism. The different regimes of sample responses are discussed in the main text and Figure 1

for short time delays $t''=t_1$, when hopping is correlated, and longer time delays $t''=t_2$, when hopping reaches the random walk limit.

The probe signal is summed across the thickness L of the sample. For mismatched group velocities between the pump and probe pulses, and for $t''< L/\Delta v_g$, where Δv_g is the mismatch of the group velocities of the pump and probe pulses[5,10–12], the probe overtakes the pump within the sample, and samples the instantaneous electronic polarization in addition to the ionic responses. For a terahertz pump and 800 nm probe in a sample mostly composed of alumina, refractive indices are taken as $n_{probe}\approx1.76$, and $n_{pump}\approx3.08$[10,13], i.e. the probe propagates faster than the pump inside the sample. Ignoring the absorption of the pump pulse, and for the probe pulse assumed short, the strength of this instantaneous electronic-origin signal is proportional to the thickness of the sample convoluted with the square of the pump field and the nonlinear refractive index of the sample[10]. In fitting the TKE signals of β-aluminas and K β''-alumina, this component is referred to as the "instantaneous component". A free constant is included as a long-time asymptote due to the finite time delays probed. This is consistent with wide distributions of hopping lifetimes[14].

Supplementary Note 2: TKE Control Measurements

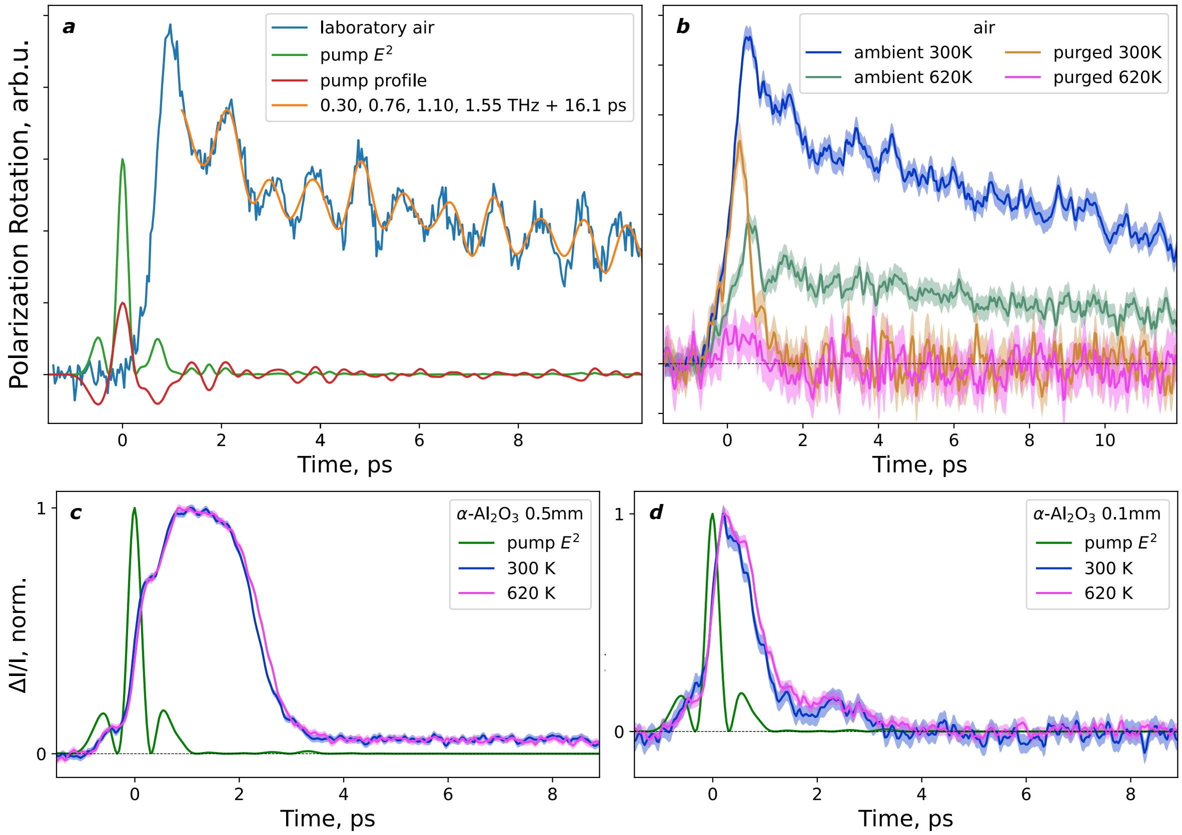

Figure S1 | Terahertz Kerr effect (TKE) in control samples. (**a**) TKE signal of ambient air (blue), and linear prediction fitting of oscillatory components (orange). (**b**) TKE signals of ambient air at 300 K (blue), purged air at 300 K (ochre), and ambient and purged air at 620 K (green and pink, respectively). The cited temperature is the temperature of the heating stage, with the pump and probe pulses passing through an opening in it. (**c**) TKE of 0.5 mm thick sapphire at 300 K (blue) and 620 K (pink). (**d**) TKE of 0.1 mm thick sapphire at 300 K (blue) and 620 K (pink). In (**b-d**), the shaded regions represent ±1 s.e. of the mean.

Figure S1 shows TKE measurements of ambient air and sapphire samples. The TKE of ambient air shows a signal with components matching the absorption lines of water vapor at ≈0.76 THz and ≈1.1 THz[15]. This signal decreases by ≈half when the absolute temperature is ≈doubled (Figure S1b), and disappears entirely when the volume of pump-probe spatial overlap is purged with dry nitrogen, independent of temperature (Figure S1b). We therefore assign this signal to the rotational coherences of gas-phase atmospheric water. The strength of the rotational coherence of atmospheric nitrogen at ≈8.4 ps (Figures 2a and 3a, main text) also decreases by approximately half, relative to the remainder of the signal, which remains approximately constant in magnitude, upon heating from 300 K to 620 K. This is consistent with previous measurements of rotational coherences in gas-phase molecules[16].

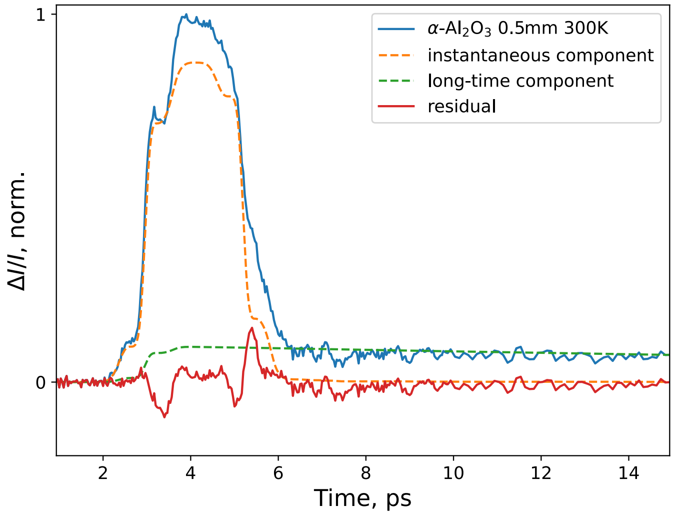

Figure S2 | TKE signal from 0.5-mm sapphire (0001) at 300 K. The TKE signal (blue) is fit to the sum of an instantaneous component (orange) and a long-time component that appears simultaneously. The residual is plotted in red. The rotational coherence of atmospheric nitrogen is at ≈11.4 ps with the peak pump field at ≈3 ps.

The TKE signals from sapphire samples (Figure S1cd) measured in purged atmosphere are temperature independent. The sapphire signal is modeled (Figure S2) as a combination of an instantaneous component (Supplementary Note 1 and Ref. [10]) and a long-time component that is absent in the 0.1-mm sample (Figure S1d). Since the long-time components ("tails") in the TKE signals of β-aluminas are, unlike for sapphire, thickness-independent, but ion- and temperature-dependent, their origin must be distinct from that of the long-time sapphire signal. A more exact model of the sapphire signals remains possible, but the simpler one suffices here to distinguish it from the responses of β-aluminas. All TKE signals scale with the peak intensity of the THz pump field (Figure S3). Whenever they are measurable, the kinetics of the relaxation are independent of the pump field strength.

The TKE signals in β-aluminas show deviations at short time delays upon prolonged (several days) measurements (Figure S4a), which can be reversed by stopping measurements. The measurements presented in the main text are taken when such effects are minimized, e.g., on fresh samples. The changes over the course of each measurement, up to ≈12 hours, are small, and the long-time-delay components of the signals are unaffected (examples in Figure S4ef) even with a stronger probe pulse. We include X-ray microdiffraction characterization of the Kβ''-alumina sample

(Figure S4b), and optical microscopy images of thin (Figure S4c) and thick (Figure S4d) samples of the β-aluminas. No phase impurities were detected in the K β"-alumina sample beyond the components of the pristine polycrystalline pellet and the inconel support.

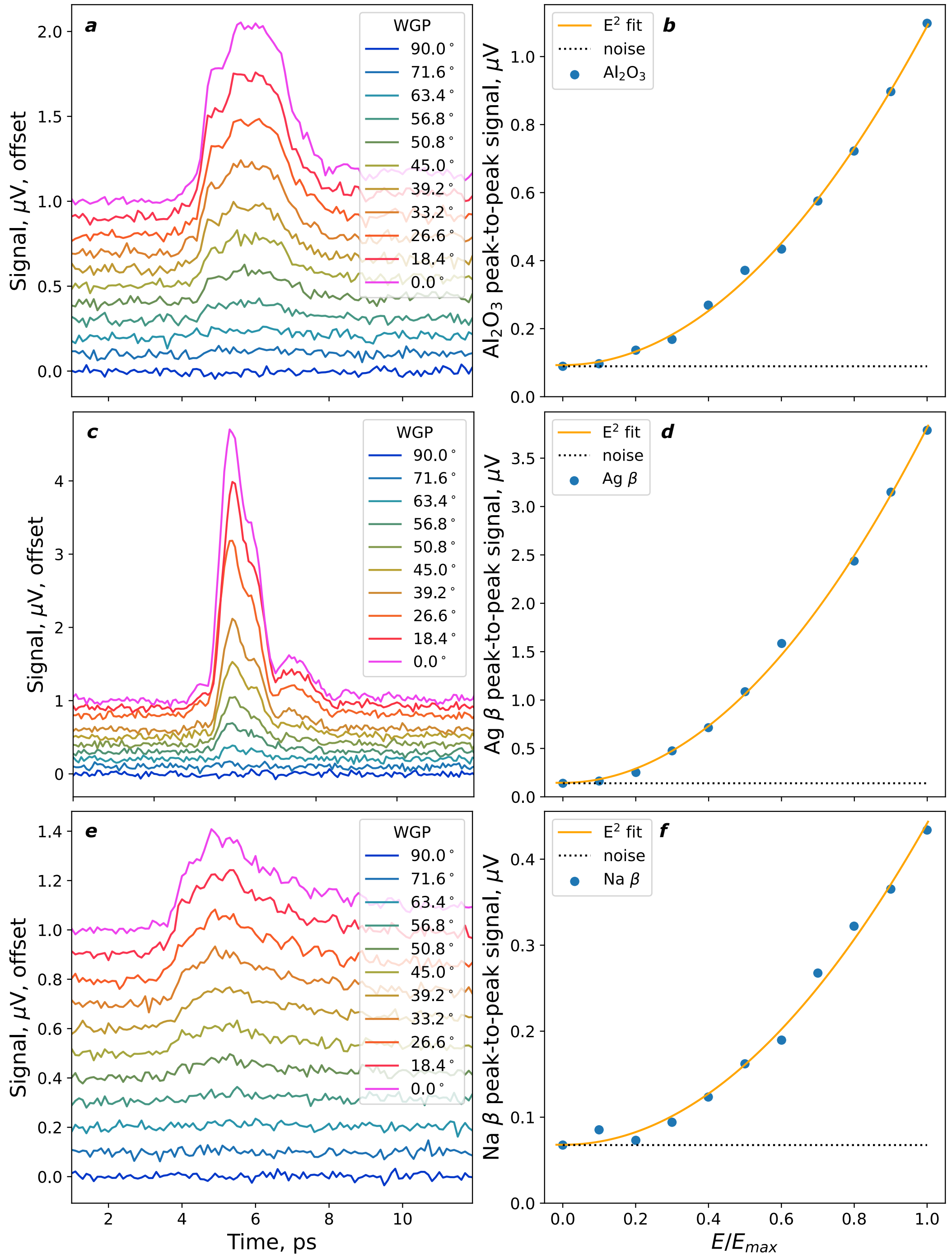

Figure S3 | Pump field dependence of TKE signals. Time-domain TKE signals as functions of wire-grid polarizers (WGP) angle for 0.5-mm sapphire (**a**), ≈0.4-mm Ag β-alumina (**c**), and ≈0.4-mm Na β-alumina (**e**). The pump field is maximum at the zero degrees position of the WGP, and the pump is fully blocked at 90 degrees. The peak-to-peak signals for sapphire (**b**), Ag β-alumina (**d**), and Na β-alumina (**f**) with fits (orange) to a noise level (dotted) and the intensity of the THz pump field.

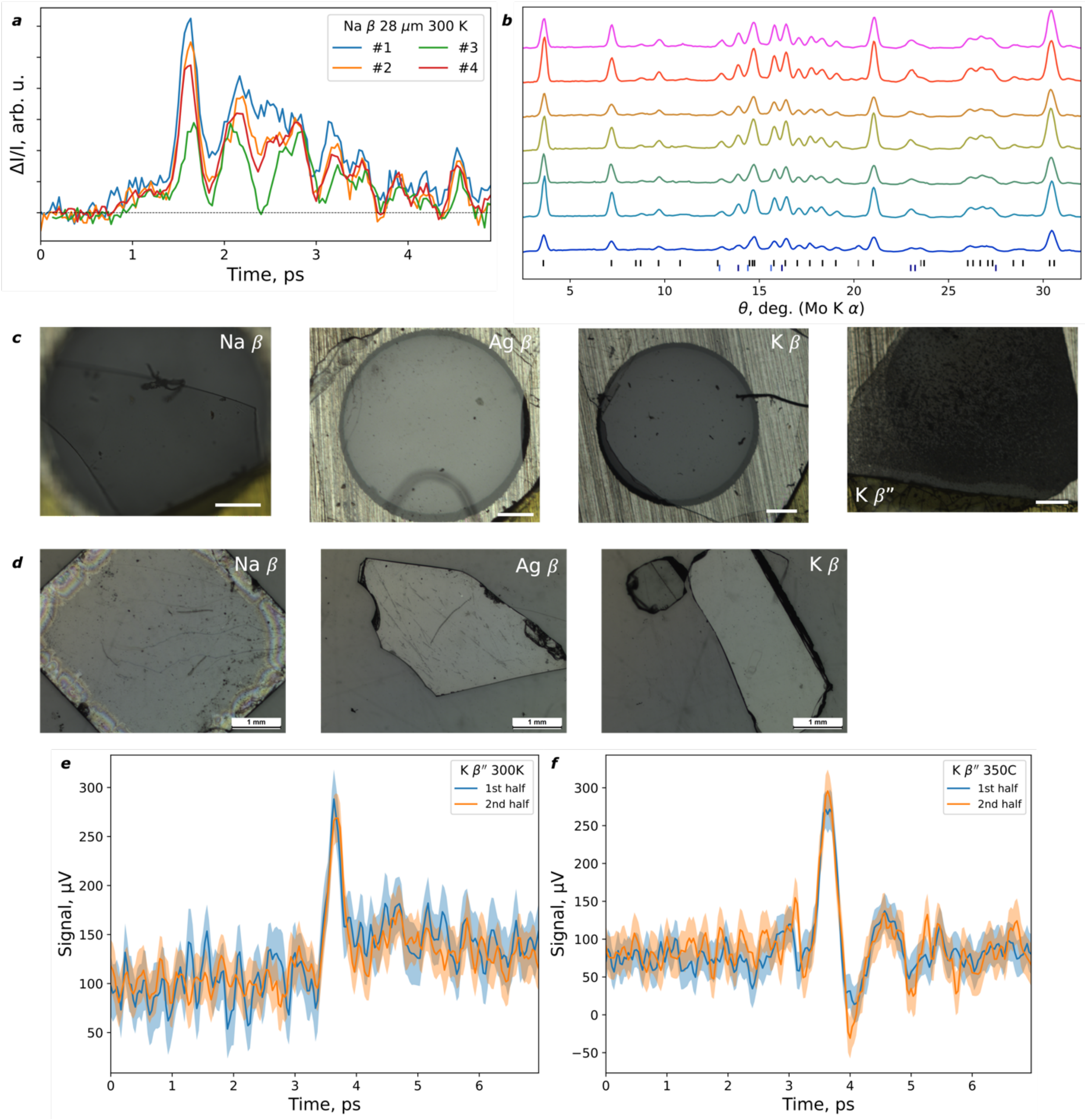

Figure S4 | Stability of the TKE signals and post-measurement characterization. (**a**) TKE in a thin sample of Na β-alumina, measured four times (#1 through #4 chronologically) over ≈4 days with other measurements in between. There was a pause between the third and fourth measurements. Here, the peak pump field is applied at 1.5 ps. (**b**) X-ray microdiffraction characterization of the measured thin sample of K β"-alumina. Color curves: seven micro-diffraction patterns collected from the polycrystalline K β"-alumina sample following TKE measurements and additional 800nm illumination. The locations of major diffraction peaks plotted along the bottom are K β"-alumina (black, ICSD 200993, with lattice parameters adjusted as in the fit reported by Baclig *et al.*[17]), cubic and tetragonal zirconia (dark blue, ICSD 66781, and light blue, ICSD 26488, respectively), and Ni_3Cr alloy (grey, COD 1525114) to represent the Inconel support on which the sample is mounted. (**c**) Optical microscope images of all thin samples mounted for TKE measurements; scale bars are 200 µm. (**d**) Optical microscope images of thicker β-alumina samples used for TKE measurements; scale bars are 1 mm. (**e,f**) TKE signals in K β"-alumina dis-aggregated by the order of sweeps within measurements at 300 K (e) and 350 C (f).

Supplementary Note 3: Optical Conductivity from THz Transmission

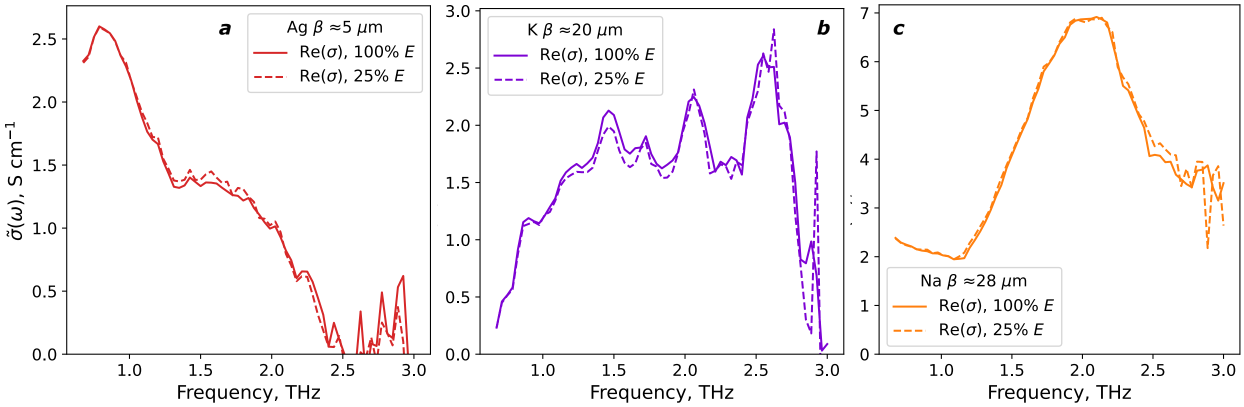

Figure S5 | Optical conductivity of *β*-aluminas in the far infrared. Real part of the optical conductivity σ, calculated from terahertz transmission through single-crystalline Ag (**a**, dark red), K (**b**, purple), and Na (**c**, orange) *β*-aluminas. The thicknesses of samples used are noted in the legends. The relative uncertainties in the absolute values due to the choice of starting fit guess are ≤50%. For each, the transmission measurements were performed at ≥500 kV/cm (denoted as 100% *E* field, solid lines) and a quarter of that (denoted as 25% *E* field, dashed lines). In all cases, the polarization of the pump field was parallel to the conduction planes of the crystals. Below ≈0.7 THz, and above ≈2.8 THz, the transmission was weak and conductivity calculations noisy.

The complex refractive index ñ(ω) was fit to the transmission at each frequency[18–20]. For a free-standing slab of thickness L, the transmission T(ω) relative to a pinhole reference is given as:

$$T(\omega) = \frac{4\tilde{n}(\omega)}{(\tilde{n}(\omega)+1)^2}\exp\big(ikL(\tilde{n}(\omega)-1)\big) \qquad \text{(S4)}$$

Here, *k* is the wavenumber equal to ω/c. This nonlinear equation was solved for ñ(ω) at each frequency provided an initial guess $\tilde{n}_0(\omega)$; the values shown in Figure S5 use $\tilde{n}_0 = 2+0.5i$. The relative uncertainty due to choices of $\tilde{n}_0$ is ≤50%, but the solver is robust to small uncertainties in other inputs, such as possible variation or e.g. 20% error in the sample thickness. This method also avoids the simplifying single-pass assumption, which may not apply at all frequencies due to strong variation in absorption across the pump spectrum.

The optical conductivities of *β*-aluminas show features and absolute magnitudes consistent with literature spectra of melt-grown crystals[21]: ≈0.8 THz in Ag *β*-alumina, 2.1 THz in Na *β*-alumina, and several modes between 1.5, 2.0, and 2.7 THz in K *β*-alumina. More importantly, for all conditions and all samples the calculated refractive index and conductivity are independent of the terahertz field for the same initial $\tilde{n}_0$ used to solve the nonlinear equation. Unlike in recent work[22], no field-strength-dependent shifting or bleaching of any features are observed over multiple measurements of each sample.

Supplementary Note 4: Terahertz Pumps in Molecular Dynamics

The selective excitation of mobile-ion motions in β-aluminas is verified with simulations including the pump field. During the simulated terahertz pump pulse, the temperature of the mobile ions, computed by LAMMPS using the kinetic energy of the ions, rises rapidly (Figure S6ac). After the pulse is turned off, the temperature of the mobile ions begins to decrease, and the temperature of the host lattice species increases. For all materials, the terahertz pulses selectively deposit energy into the mobile ions, which then thermalize with the lattice on a picosecond timescale. This thermalization is slower than that of the relaxation of anisotropy in hopping, highlighting the selectivity of the nonlinear optical measurement to the directional alignment of ionic hopping.

The excitation of vibrational modes by the pump pulse, in addition to hopping, is verified by tracking the displacements of the center of mass of the mobile ions, projected onto the direction of the field, and referenced to the host-lattice displacement[23]. Simplifying the ensembles of mobile ions and the host lattice to a pair of point charges, the emitted field from coherent oscillations excited by the pump should be proportional to the velocity of the relative center-of-mass motions. This yields several material-specific vibrations (Figure S7) with frequencies typically within 10-20% of known literature infrared- and Raman-active vibrations[21,24–30]. For example, simulated vibrations are 0.6 and 1.0 THz in Ag β-alumina, and 1.7 THz in Na β-alumina. For simulated K β-alumina, the vibration at ≈1.3 THz is only excited at 300 K, while the vibration at ≈2.0 THz is excited at both 300 K and 600 K, in agreement with the TKE experiment (main text Figure 2b). For a classical simulation that does not account for partial covalency, this is excellent agreement. The simulated coherent displacements of the mobile-ion center of mass are between 5-15 picometers during the application of the pulse, in line with terahertz-frequency pump-probe studies in solid-state materials[5,6,31].

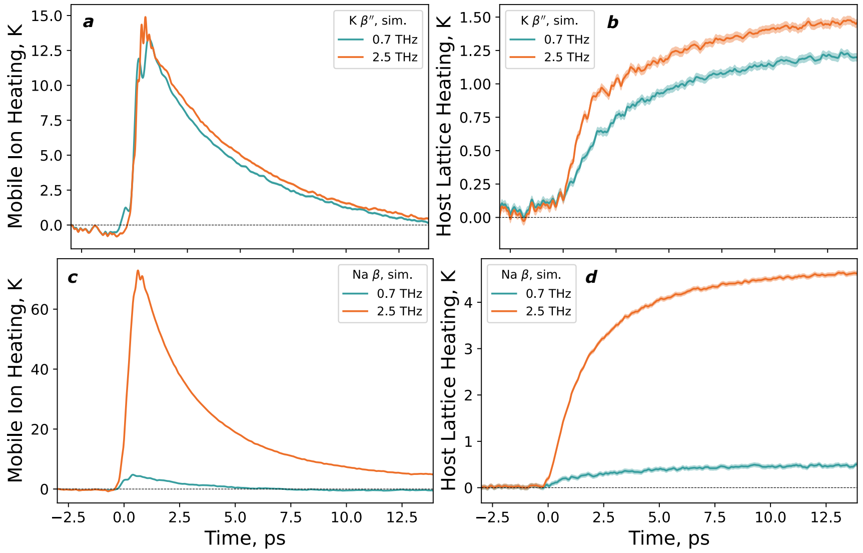

Figure S6 | Heating of β"- and β-aluminas by simulated terahertz pulses. (a,b) Simulated temperature rise in K β"-alumina: mobile ions (a) and the host lattice (b) with electric field pulses at 0.7 THz (teal) and 2.5 THz (orange). (c,d) Simulated temperature rise in Na β-alumina: mobile ions (c) and the host lattice (d) with electric field pulses at 0.7 THz (teal) and 2.5 THz (orange). The shaded areas are ±1 s.e. of the mean.

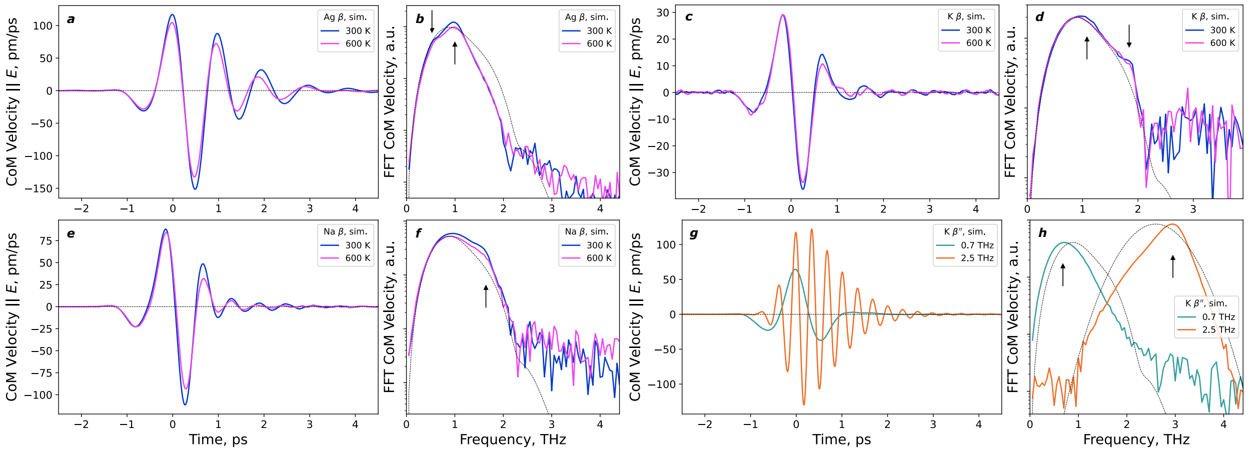

Figure S7 | Simulated excitation of coherent motions of mobile ions by terahertz pumps. Velocities of the center-of-mass of all mobile ions (**a**,**c**,**e**,**g**), and their Fourier transforms (**b**,**d**,**f**,**h**) plotted on logarithmic scales. Dashed black lines in the Fourier transform figures are spectra of the applied pump pulses. Highlighted vibrations: 0.6 and 1.0 THz in Ag β-alumina (**a**,**b**), 1.3 and 2.0 THz in K β-alumina (**c**,**d**), 1.7 THz in Na β-alumina (**e**,**f**), and 3.0 THz in K β"-alumina (**g**,**h**).

The *in silico* TKE of β-aluminas is dis-aggregated by the chemical environment of the mobile ions: (a) ions bound in a defect cluster, and (b) ions that are free to diffuse. The cluster consists of four mobile ions on six crystallographic lattice sites around an oxygen interstitial[14,32,33]. These ions shift between the sites within the cluster. The hopping components of *in silico* TKE of Na β-alumina (Figure S8) are similar at short time delays (Figure S8bc) – but only the un-bound ions yield anisotropic hopping past 1 ps (Figure S8b). This response is also weakly thermally activated.

The temperature-independence of the hopping anisotropy response due to cluster-bound ions complicates the interpretation of experimental TKE measurements of Na and Ag β-aluminas (Figure 1cd). Further, the relaxation time constants are faster than in K β-alumina (Extended Data Figures 3-5). By analogy with high-frequency NMR relaxation[34,35], the temperature activation of the back-hopping in Na and Ag β-aluminas is expected to be weaker than in K β-alumina. We focus on the K β-alumina signals because they possess stronger activation, more distinguishable time constants, and simpler interpretation due to the lack of a simulated response from the defect clusters in that material. At the same time, the agreement between the classical molecular dynamics simulation of K β-alumina and the low-frequency conductivity is the worst of all β-aluminas[14]. We believe this is due to the inability of the simulation to accurately describe the two-coordinate K environment at the anti-Beevers-Ross sites, which leads to an overestimation of activation energy and overall lower conductivity. This is consistent with the pumped molecular dynamics simulations also under-estimating the lifetimes of ions in high-energy sites and predicting a faster than experimental relaxation (Figure 3). However, the qualitative experiment-simulation agreement spans the existence of the hopping anisotropy and its temperature-, frequency- and material-dependences. A more quantitative correspondence between simulation and experiment will require *ab initio* methods. We note that our computational approach of simulating experimental impulsive pumps is distinct from the computational partitioning of energy into pre-selected vibrational modes in a recent study by Gordiz *et al.*[36]

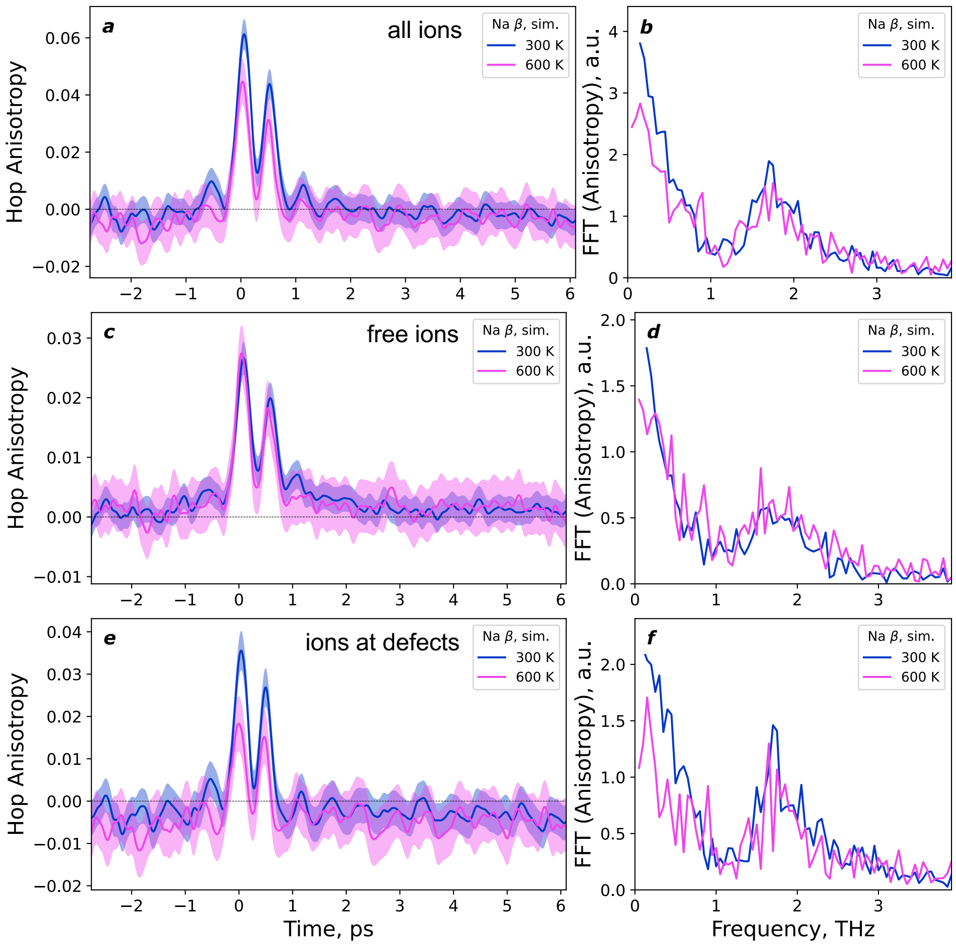

Figure S8 | Temperature dependence of the simulated hopping anisotropy in Na *β*-alumina. (**ab**) all mobile ions, (**cd**) mobile ions away from defects, i.e. “free”, and (**ef**) mobile ions within defect clusters. The shaded areas are ±1 s.e. of the mean.

Supplementary Note 5: Logical and Thermodynamic Reversibility

Here, we draw a brief analogy to concepts and experiments in stochastic thermodynamics. The parallels between memory, correlations, and the thermodynamics of information have been formulated in multiple frameworks, and we direct the reader to several reviews[37–40]. In Figure S9, we highlight three scenarios for the dissipation of terahertz pump energy that drives ionic hopping with varying degree of coupling to transport.

In the first case, the pump drives hopping between two lattice sites (illustrated as $B \rightarrow A$, Figure S9a). However, the subsequent hopping only returns the ions to their original sites via reverse hops. In this case, no diffusion happens. The macroscopic experiment constitutes an ensemble average over many realizations of driving a particle within a double-well potential coupled to a thermal bath. The pump performs work on the system to drive this process in finite time and surmount kinetic barriers and possibly a thermodynamic energy difference between the sites. In the second step, the system completes the reverse process: the energy imparted by the pump is thermalized to the bath. The sequence $B \rightarrow A \rightarrow B$ comprises a thermodynamic cycle between two states. However, the ensemble measurement of the bulk sample is not equivalent to an average over individual ionic paths that is necessary to apply Crooks’ theorem[41–43] or the

Jarzynski equality[43–45]. While our experiment probes the rates of hopping following the pump, and not the work done by the system, the broad and non-resonant nature of this distribution is consistent with a distribution of work values. This case corresponds most closely to the higher-frequency pump of Na β-alumina discussed below (Supplementary Note 6) that drives hopping by ions that are not part of ion pairs and predominantly relax by returning hops.

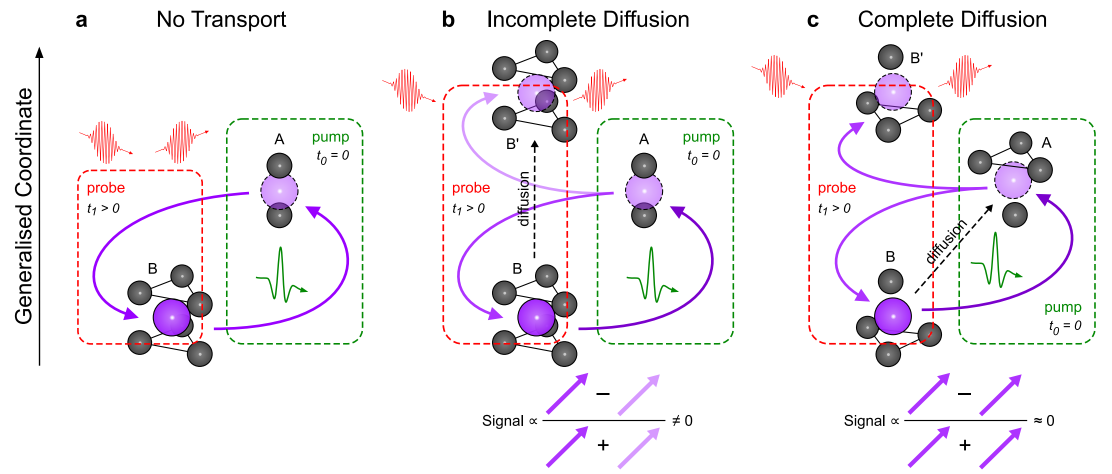

Figure S9 | Dissipation of Pump Energy to Drive Transport. **a**, Ions are driven from site B to a different site A and return without coupling to transport. **b**, Ions are driven as in **a**, and some hop on to an equivalent site B', partially completing a step of transport. **c**, Ions are driven from site B to an equivalent site A, completing a step of random-walk transport; subsequent hops to B or B' are random.

In the second case (Figure S9b), the system relaxes via two competing pathways, A $\rightarrow$ B as above, and A $\rightarrow$ B' to a site B' that is crystallographically equivalent to B but in a neighboring unit cell of the crystal. The second pathway couples the full material's network of sites and allows for the pump to ultimately drive net displacement and diffusion. Since B and B' are constructed as crystallographically equivalent, the path B $\rightarrow$ A $\rightarrow$ B' constitutes diffusion (Figure S9b), analogous to the turn of a ratchet. To measure work, sites B and B' must be distinguished, e.g., by some symmetry breaking, such as by imposing ion-permeable electrical contacts.

Assuming ideal contacts, the paths B $\rightarrow$ A $\rightarrow$ B and B $\rightarrow$ A $\rightarrow$ B' differ in the net current extractable from the pump pulse: only the latter yields a net displacement of ions and therefore current. While both pathways thermalize the pump pulse when averaged over the sample (thermodynamic irreversibility), the degree of freedom that characterizes net work (B vs B') is modified only for a fraction of the ions, with the success rate of the pathway B $\rightarrow$ A $\rightarrow$ B'. This parallels the incomplete erasure of a memory bit if the work input is too low[46–48] and suggests that higher pump fields may drive B $\rightarrow$ A $\rightarrow$ B' directly. The B $\rightarrow$ A $\rightarrow$ B' pathway mediates logical irreversibility: following the experiment, an ion that started at B' and did not move is indistinguishable from one that started at B and underwent a net displacement. By contrast, ions driven to site A by the pump are, for a time, distinguishable from the other ions at A: they are more likely to hop in a particular direction (namely, back to B) rather than randomly. In other words, for an ion at A, position alone is insufficient to determine subsequent dynamics. This insufficiency establishes a correlated hopping mechanism and distinguishes transport in this case from a random walk. The correlation between subsequent hopping and the pump-driven B $\rightarrow$ A hop is information

that dissipates over time. An example of this case is the experimental pumping of *β*-aluminas; due to the predominance of an ion-pair pathway of hopping (Figure S10 below), both rates are non-negligible. Within ion-pair hopping, the memory component can be conceptualized as the (non-)random association of an ion, e.g. at site A, with one of its neighbors.

The TKE experiment measures velocities and alone does not quantify the numbers of ions that follow the two paths, only their difference. In absence of ion-permeable contacts, and with the diameter of the pump pulse (≈0.5 mm) larger than that of the probe (<0.1 mm), the long-range restoring force that eventually reverses B' to B is slow and not measurable with TKE. Therefore, TKE does not directly quantify the success rate for information erasure. We rely on molecular dynamics simulations and on the *β''*-alumina experiment (Figure 4) to distinguish the *β*-alumina experiments, represented by the case of Figure S9b, from the fully reversible Figure S9a. This is done by verifying that some pump-driven hops indeed contribute to the B → A → B' pathway.

The third case (Figure S9c) holds if lattice sites are equivalent, and ordering is absent. Then one hop, e.g., B → A in Figure S9c, can constitute the complete diffusion process, if velocity or mean-square-displacement correlations do not carry additional information and decay on the timescale of the pump pulse. This is the case for K *β''*-alumina at 600 K, which can be represented by a random walk. In this case, the probabilities of the pathways B → A → B and B → A → B' (in Figure S9c) are the same following the pump pulse as without it, pump-driven hops are sufficient for diffusion, and no long-time TKE signal is possible. Unlike in Figure S9b, here the ions undergoing pump-driven hops B → A are already indistinguishable from those that started at A, and pump-driven hops constitute DC current if perfect contacts were available to measure it.

As the *β*- and *β''*-aluminas have been the classical model systems of solid-state ionics, a variety of high-frequency measurements of electrical[49–51], optical[52–56], scattering[30,57,58], and spin[34,35,59–61] responses have been carried out. These have been partially systematized by Kamishima[54]: the activation energy increases with decreasing frequency. Our simulations[14] and experiments (this work) highlight a corresponding timescale-dependence of the correlations in ionic hopping: the complete process of conduction is measured only at timescales slower than the persistence of such correlations. Our measurements here suggest that this effect is not due to distributions of frozen potential-energy barriers in a dilute-carrier system, but to the intrinsically fluctuating stochastic dynamics of ion motions in a non-dilute, interacting one.

Supplementary Note 6: Attempt Frequencies in Na *β*-alumina

Here, we seek to understand the fundamental initiation of ion transport, the attempt frequency v_0 for ionic hopping. In the random-walk framework (full entropy produced with every hop), v_0 is not an atomistic vibration, but an effective quantity with dimensions of frequency relating to the vibrational states at the saddle point of a generalized potential energy surface[62–64], which together with the difficulty of measurement has led to the use of averages over the density of states for its proxies[65–68]. However, even for a material with a simple power spectrum, the vibrational modes Q_i may couple to hopping rates H unequally (main text and Figure 4c). The response of *β*-aluminas to electrical fields driving hops is more complex than that of K *β*"-alumina (Figure 4c) due to the presence of defect clusters and non-equivalent crystallographic sites (Figure 1a). We first analyze steady-state simulations, followed by *in silico* TKE molecular dynamics second. We focus on the hops into the high-energy anti-Beevers-Ross (aBR) sites from the low-energy Beevers-Ross (BR) sites. As chemical doping furthermore creates distinct chemical environments for the mobile ions,

here we simulate the compositional $Na_{1+2x}Al_{11}O_{17+x}$ series for Na_2O doping x = 0,0.01,0.1, where the practical material corresponds to $x \approx 0.1$.

For a molecular dynamics simulation, we partition the trajectories of mobile ions (typically, 100 ns for steady-state simulations) into hopping events, defined as the time point when an ion migrates between crystallographic lattice sites, and the residence times between them. A residence time is defined as the period of time that an ion spends at a lattice site following a hop into that site[14]. Here, we quantify the distributions of hopping residence times in the short-time, picosecond regime. If an ion is found to preferentially hop at some (short) residence times corresponding to a multiple of some well-defined period, then such a period is empirically the inverse of the attempt frequency for hopping. For any crystallographic site within the material, the times that elapse between an ion leaving it, and a new ion entering the site, "filling times" for short, can be analyzed with this statistical method.

For the defect-free material, x = 0 (Figure S10a, blue), distributions of "filling times" for high-energy aBR sites show that hops into the sites cluster at periods of time corresponding to 2.3 THz (Figure S10b, blue), which is a minuscule yet distinct part of the Na vibrational density of states (Figure S10d, blue) and the main feature of the infrared conductivity (Figure S10e, blue). We conclude that 2.3 THz is the attempt frequency for BR $\rightarrow$ aBR hopping in absence of neighboring Na ions already occupying aBR sites. This matches the experimentally measured infrared-active vibration at 2.0-2.1 THz (Supplementary Note 3). Adding even a single defect cluster per 100 formula units (x = 0.01) increases the rates of hopping (Figure S10a, ochre) and already changes the frequency makeup of the hopping attempts towards lower frequencies (Figure S10b, ochre). The additional Na ion locates on the high-energy sites, and necessarily perturbs the energetics of its neighbors, resulting in interstitialcy, or ion-pair, diffusion[69] that originates with perturbed, softer attempt frequencies due to the repulsion between mobile ions. For the practical simulated stoichiometry, x = 0.1, the distribution of site filling times shows a longer period at short times (Figure S10a, pink), which corresponds to the practical attempt frequency of 1.4±0.2 THz with some variation across the sites (Figure S10c). This vibration is the empirical attempt frequency for interstitialcy knock-on hopping in the practical Na β-alumina, with the simulated frequency corresponding to one measurable with inelastic neutron scattering at 5-6 meV (1.2-1.4 THz)[57,70]. The simulated vibrations at 1.4±0.2 and 2.3 THz (5-6 meV and 2.1 THz experimentally), are attempt frequencies for hopping from two distinct states (Figure S10f) via two mechanisms: interstitialcy ion-pair and solo, respectively. While both are present in the real material, most transport occurs via the interstitialcy or ion-pair pathway.

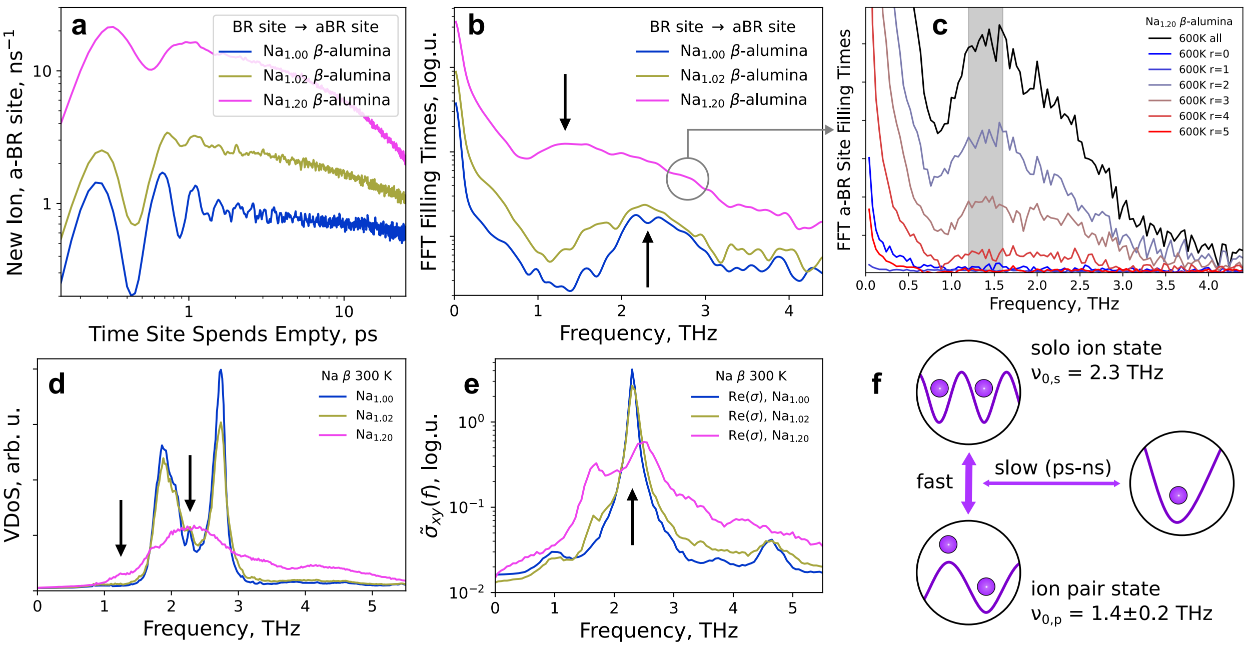

Figure S10 | Attempt frequencies for Na β-alumina from MD simulations. (**a**) Distributions of times that anti-Beevers-Ross sites spend empty following a Na ion hopping away, simulated at 600 K for x = 0,0.01,0.10 (blue, ochre, and pink, respectively) in stoichiometries $Na_{1+2x}Al_{11}O_{17+x}$. (**b**) Fourier transforms of the distributions in (**a**), smoothed with a Gaussian filter of 0.1 THz std. dev. Arrows highlight peaks at 2.3 THz and 1.4 THz corresponding to the short-time structure of the distributions in (**a**). (**c**) Fourier transforms of the x = 0.10 distribution in (**a,b**) dis-aggregated by the distances from sites to O_i interstitials: "r = 0" is immediately adjacent to defects (see Supplementary Note 5 and Figure S7a of Ref. 14 for more details), and black is the total distribution. (**d**) Na vibrational density of states and (**e**) real part of the optical conductivity σ for the three simulated stoichiometries of Na β-alumina, with arrows highlighting the same frequencies as in (**b**). (**f**) Model of the three internal states for mobile ions in Na β-alumina: solo as in the defect-free (x = 0) material, part of a mobile-ion pair, and part of a defect cluster. The first two states possess distinct attempt frequencies for hopping, 2.3 THz and 1.4±0.2 THz, respectively, whereas the defect cluster is non-diffusive.

Both vibrations are distinct from the LO phonon mode at 3.0 THz detectable by TKE (Figure 2), OKE (Extended Data Figure 6), and neutron scattering[57]. The LO phonon was previously thought to correlate to hopping and activation energy[71–73]. The 1.4±0.2 THz attempt frequency corresponds to the interstitialcy nearest-neighbor mobile-ion pair, i.e., a defect created by chemical doping, whereas the 2.3 THz frequency is the attempt frequency for BR → aBR hopping in absence of nearby Na ions already in aBR sites (Figure S10f). This softening of the attempt frequency with doping demonstrates the role of repulsions between mobile ions in driving ionic conductivity. Indeed, chemical doping alters not only the concentrations of the active species, but also the very energetics of each hop. Finally, the practical attempt frequency of 1.4±0.2 THz comprises only a minor part of both the Na vibrational density of states (Figure S10d) and simulated Na optical conductivity (Figure S10e). By contrast, the optical phonon at 3.0 THz does not contribute to hopping, the same way that higher-frequency modes in K β"-alumina also do not contribute (main text, Figure 4c). Overall, our statistical analysis enables a detailed examination of the vibrational contributions to hopping.

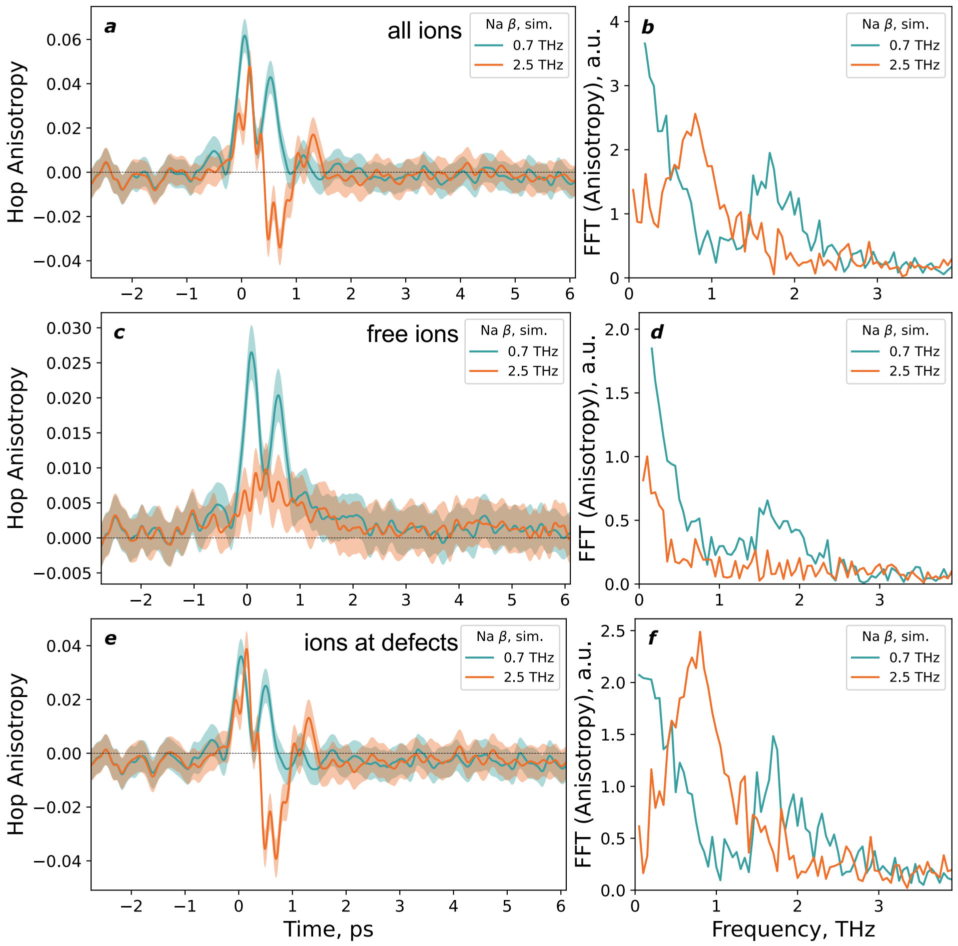

Figure S11 | Simulated anisotropy of hopping in Na *β*-alumina at 300 K. Time traces with the peak applied fields at zero time (**ace**), and Fourier transforms of the hopping anisotropy (**bdf**). Two pump pulses are compared: 0.7 THz (teal) repeated from Figure S8, and 2.5 THz (orange). The full response (**ab**) is disaggregated to the response of unbound ions (**cd**) and ions bound within defect clusters (**ef**). The shaded areas are ±1 s.e. of the mean.

We now simulate an *in silico* TKE experiment for Na *β*-alumina, at the practical stoichiometry x = 0.1 and 300 K. Given two distinct mechanisms of hopping (Figure S10f) with two attempt frequencies, each could yield hopping when pumped. As for K *β*”-alumina (main text Figure 4c), pump field frequencies of 0.7 THz and 2.5 THz are simulated at peak fields 300 kV/cm inside the material. The simulated 2.5-THz pulse raises the temperature of the system by ≈4.5 K, 10× more than the 0.7 THz pulse at ≈0.45K (Figure S6cd). The hopping anisotropy in response to the simulated pumps (Figure S11) depends strongly both on the pumping frequency and on the location of the mobile ion (cluster-bound vs free). The response of free ions to the simulated 2.5-THz pulse is distinct from zero, but relatively weak given the strong absorption via the 2.3 THz mode. This is consistent with the solo hopping mechanism with the 2.3-THz attempt frequency providing only a minor contribution in the practical material. Finally, the overall hopping anisotropy response is strongly convoluted by defect clusters (Figure S11c). Overall, our simulation shows that TKE results at short time delays should be interpreted with care for materials with multiple possible hopping mechanisms and internal states. Knowledge of the local

environments of mobile ions is required to rigorously characterize the possible attempt frequencies in ionic conductors. We focus on the K β"-alumina in the main text for simplicity.

We further dis-aggregate the simulated anisotropy of hopping by the site of origin for each hop for Na and K β-aluminas, as shown in Figure S12. Notably, hops from both types of sites show a similar long-lived anisotropy due to the predominant excitation ion-pair hopping by the 0.7-THz pump. When a 2.5-THz pump is simulated (Figure S12ef, orange), the site-specific traces are markedly different. The BR → aBR hopping is excited first, followed by aBR → BR.

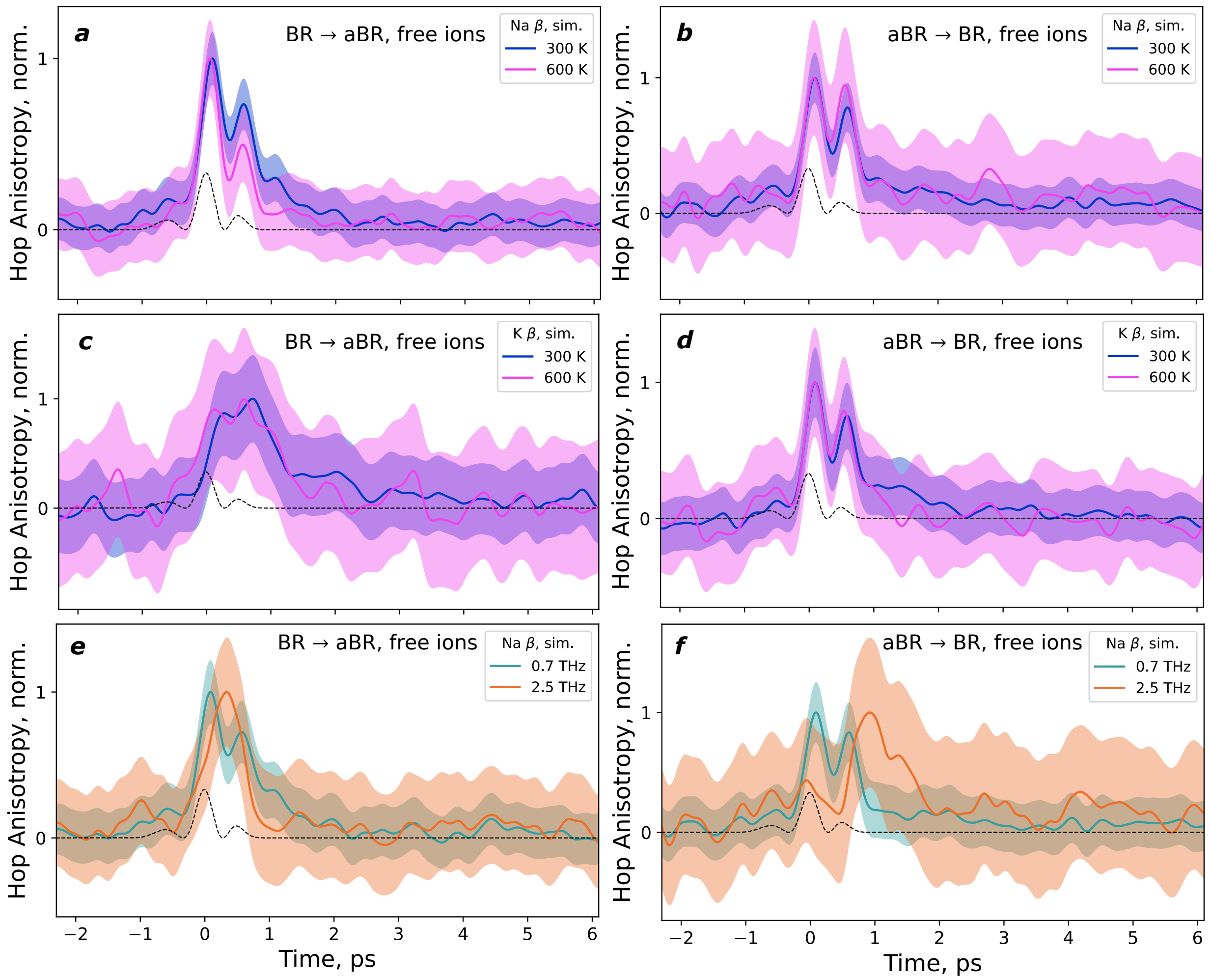

Figure S12 | Dependence of the simulated hopping anisotropy in β-aluminas on site type and pumping frequency. (**ace**) hops from Beevers-Ross (BR) sites to anti-Beevers-Ross (aBR) sites. (**bdf**) hops from anti-Beevers-Ross (aBR) sites to Beevers-Ross (BR) sites. (**a**,**b**) Na, (**c**,**d**) K, (**a**,**b**) Na for two pumping frequencies: 0.7 THz (teal) and 2.5 THz (orange) at 600 K. Shaded regions represent ±1 s.e. of the mean. Only sites away from defect clusters are used.

Table S1: high-frequency activation energies from NMR relaxation rates and high-frequency conductivity measurements (denoted as GHz). The absence of an uncertainty value for Ref. 74 reflects that the value is taken from Ref. 74 directly rather than from our own fitting, and uncertainty is not reported there. The absence of uncertainty values for Ref. 49 reflects the lack of clarity on the co-variation of data reported there (see their Figure 5), or our use of their reported values. The absence of uncertainty values for Ref. 75 reflects the paucity of data points. Otherwise, our fitting uncertainty values are ±1 s.e.

**Material**	**Method**	**Frequency, MHz**	**Low T E_A, meV**	**High T E_A, meV**	**Ref.**
Na β-alumina, melt	NMR	17.2	30±2	181±15	[34]
Na β-alumina, melt	NMR	25.5	40±2	105±4	[34]
Na β-alumina, melt	NMR	21	42±2	135±4	[59]
Na β-alumina	NMR	5.2	39	N/A	[74]
Na β-alumina, melt	GHz	1200	41	118	[49]
Na β-alumina	GHz	9460	N/A	83±4	[76]
Na β-alumina, melt	GHz	24000	66		[49]
Ag β-alumina	NMR	400	28±1	98±5	[35]
Ag β-alumina	GHz	380	24, 54	145	[49]
Ag β-alumina	GHz	24000	N/A	110	[49]
K β-alumina	NMR	11.1	60±4	N/A	[61]
K β-alumina	GHz	1200	N/A	192	[49]
K β-alumina	GHz	24000	N/A	116	[49]
Na β"-alumina	NMR	39.6	39±5, 98±7	252±9	[60]
Na β"-alumina	GHz	18000	30	199	[75]
Na β"-alumina	GHz	60000	29	N/A	[75]

Supplementary References

1. Finneran, I. A. *et al.* 2D THz-THz-Raman Photon-Echo Spectroscopy of Molecular Vibrations in Liquid Bromoform. *J. Phys. Chem. Lett.* **8**, 4640–4644 (2017).
2. Finneran, I. A. *et al.* Coherent two-dimensional terahertz-terahertz-Raman spectroscopy. *Proc. Natl. Acad. Sci.* **113**, 6857–6861 (2016).
3. Savolainen, J., Ahmed, S. & Hamm, P. Two-dimensional Raman-terahertz spectroscopy of water. *Proc. Natl. Acad. Sci.* **110**, 20402–20407 (2013).
4. Ciardi, G., Berger, A., Hamm, P. & Shalit, A. Signatures of Intra- And Intermolecular Vibrational Coupling in Halogenated Liquids Revealed by Two-Dimensional Raman-Terahertz Spectroscopy. *J. Phys. Chem. Lett.* **10**, 4463–4468 (2019).
5. von Hoegen, A., Mankowsky, R., Fechner, M., Först, M. & Cavalleri, A. Probing the interatomic potential of solids with strong-field nonlinear phononics. *Nature* **555**, 79–82 (2018).
6. Kozina, M. *et al.* Terahertz-driven phonon upconversion in $SrTiO_3$. *Nat. Phys.* **15**, 387–392 (2019).
7. Kryvohuz, M. & Mukamel, S. Multidimensional measures of response and fluctuations in stochastic dynamical systems. *Phys. Rev. A.* **86**, 1–12 (2012).
8. Ikeda, T., Ito, H. & Tanimura, Y. Analysis of 2D THz-Raman spectroscopy using a non-Markovian Brownian oscillator model with nonlinear system-bath interactions. *J. Chem. Phys.* **142**, (2015).
9. Elgabarty, H. *et al.* Energy transfer within the hydrogen bonding network of water following resonant terahertz excitation. *Sci. Adv.* **6**, 1–15 (2020).
10. Sajadi, M., Wolf, M. & Kampfrath, T. Terahertz-field-induced optical birefringence in common window and substrate materials. *Opt. Express* **23**, 28985 (2015).

11. Maehrlein, S. F. *et al.* Decoding ultrafast polarization responses in lead halide perovskites by the two-dimensional optical Kerr effect. *Proc. Natl. Acad. Sci.* **118**, e2022268118 (2021).
12. Huber, L., Maehrlein, S. F., Wang, F., Liu, Y. & Zhu, X. Y. The ultrafast Kerr effect in anisotropic and dispersive media. *J. Chem. Phys.* **154**, (2021).
13. Grischkowsky, D., Keiding, S. S., van Exter, M. & Fattinger, C. Far-infrared time-domain spectroscopy with terahertz beams of dielectrics and semiconductors. *J. Opt. Soc. Am. B* **7**, 2006 (1990).
14. Poletayev, A. D., Dawson, J. A., Islam, M. S. & Lindenberg, A. M. Defect-driven anomalous transport in fast-ion conducting solid electrolytes. *Nat. Mater.* **21**, 1066–1073 (2022).
15. van Exter, M., Fattinger, C. & Grischkowsky, D. Terahertz time-domain spectroscopy of water vapor. *Opt. Lett.* **14**, 1128 (1989).
16. Fleischer, S., Zhou, Y., Field, R. W. & Nelson, K. A. Molecular orientation and alignment by intense single-cycle THz pulses. *Phys. Rev. Lett.* **107**, 1–5 (2011).
17. Baclig, A. C. *et al.* High-Voltage, Room-Temperature Liquid Metal Flow Battery Enabled by Na-K|K-β''-Alumina Stability. *Joule* **2**, 1287–1296 (2018).
18. Neu, J. & Schmuttenmaer, C. A. Tutorial: An introduction to terahertz time domain spectroscopy (THz-TDS). *J. Appl. Phys.* **124**, (2018).
19. Spies, J. A. *et al.* Terahertz Spectroscopy of Emerging Materials. *J. Phys. Chem. C* **124**, 22335–22346 (2020).
20. Morimoto, T. *et al.* Microscopic ion migration in solid electrolytes revealed by terahertz time-domain spectroscopy. *Nat. Commun.* **10**, (2019).
21. Allen, S. J., Cooper, A. S., Derosa, F., Remeika, J. P. & Ulasi, S. K. Far-infrared absorption and ionic conductivity of Na, Ag, Rb, and K β-alumina. *Phys. Rev. B* **17**, 4031–4042 (1978).
22. Minami, Y. *et al.* Macroscopic Ionic Flow in a Superionic Conductor Na+ β-Alumina Driven by Single-Cycle Terahertz Pulses. *Phys. Rev. Lett.* **124**, 147401 (2020).
23. Marcolongo, A. & Marzari, N. Ionic correlations and failure of Nernst-Einstein relation in solid-state electrolytes. *Phys. Rev. Mater.* **1**, 025402 (2017).
24. Colomban, P., Mercier, R. & Lucazeau, G. Vibrational study of and conduction mechanism in β alumina. II. Nonstoichiometric β alumina. *J. Chem. Phys.* **75**, 1388–1399 (1981).
25. Colomban, P. & Lucazeau, G. β″-and ion-rich β-alumina: Comparison of vibrational spectra and conductivity parameters. *Solid State Ionics* **2**, 277–288 (1981).
26. Hao, C. H., Chase, L. L. & Mahan, G. D. Raman scattering in beta-alumina. *Phys. Rev. B* **13**, 4306–4313 (1976).
27. Chase, L. L., Hao, C. H. & Mahan, G. D. Raman scattering from sodium and silver in β-alumina. *Solid State Commun.* **18**, 401–403 (1976).
28. Klein, P. B., Schafer, D. E. & Strom, U. Cation interstitial pair modes in the vibrational spectra of mixed β-aluminas. *Phys. Rev. B* **18**, 4411–4421 (1978).
29. Barker, A. S., Ditzenberger, J. A. & Remeika, J. P. Lattice vibrations and ion transport spectra in β-alumina. I. Infrared spectra. *Phys. Rev. B* **14**, 386–394 (1976).
30. Lucazeau, G. Infrared, Raman and neutron scattering studies of β- and β″-alumina: a static and dynamical structure analysis. *Solid State Ionics* **8**, 1–25 (1983).
31. Neugebauer, M. J. *et al.* Comparison of coherent phonon generation by electronic and ionic Raman scattering in $LaAlO_3$. *Phys. Rev. Res.* **3**, 013126 (2021).
32. Wolf, D. On the mechanism of diffusion in sodium beta alumina. *J. Phys. Chem. Solids* **40**, 757–773 (1979).
33. Zendejas, M. A. & Thomas, J. O. Conduction mechanisms in solid electrolytes: Na^+ beta-alumina. *Phys. Scr.* **1990**, 235–244 (1990).
34. Walstedt, R. E., Dupree, R., Remeika, J. P. & Rodriguez, A. ^{23}Na nuclear relaxation in Na β-alumina: Barrier-height distributions and the diffusion process. *Phys. Rev. B* **15**, 3442–3454 (1977).
35. Iwai, Y., Kamishima, O., Kuwata, N., Kawamura, J. & Hattori, T. ^{109}Ag NMR and relaxation

mechanism in single crystal Ag β-alumina. *Solid State Ionics* **179**, 862–866 (2008).
36. Gordiz, K., Muy, S., Zeier, W. G., Shao-Horn, Y. & Henry, A. Enhancement of ion diffusion by targeted phonon excitation. *Cell Reports Phys. Sci.* **2**, 100431 (2021).
37. Parrondo, J. M. R., Horowitz, J. M. & Sagawa, T. Thermodynamics of information. *Nat. Phys.* **11**, 131–139 (2015).
38. Jarzynski, C. Equalities and Inequalities: Irreversibility and the Second Law of Thermodynamics at the Nanoscale. *Annu. Rev. Condens. Matter Phys.* **2**, 329–351 (2011).
39. Ciliberto, S. Experiments in Stochastic Thermodynamics: Short History and Perspectives. *Phys. Rev. X* **7**, 16–21 (2017).
40. Ciliberto, S. & Lutz, E. The Physics of Information: From Maxwell to Landauer. in *Energy Limits in Computation* 155–175 (Springer International Publishing, 2019).
41. Crooks, G. E. Nonequilibrium measurements of free energy differences for microscopically reversible Markovian systems. *J. Stat. Phys.* **90**, 1481–1487 (1998).
42. Collin, D. *et al.* Verification of the Crooks fluctuation theorem and recovery of RNA folding free energies. *Nature* **437**, 231–234 (2005).
43. Liphardt, J., Dumont, S., Smith, S. B., Tinoco, I. & Bustamante, C. Equilibrium information from nonequilibrium measurements in an experimental test of Jarzynski's equality. *Science.* **296**, 1832–1835 (2002).
44. Jarzynski, C. Nonequilibrium equality for free energy differences. *Phys. Rev. Lett.* **78**, 2690–2693 (1997).
45. Jarzynski, C. Equilibrium free-energy differences from nonequilibrium measurements: A master-equation approach. *Phys. Rev. E* **56**, 5018–5035 (1997).
46. Bérut, A. *et al.* Experimental verification of Landauer's principle linking information and thermodynamics. *Nature* **483**, 187–189 (2012).
47. Gavrilov, M., Chétrite, R. & Bechhoefer, J. Direct measurement of weakly nonequilibrium system entropy is consistent with Gibbs–Shannon form. *Proc. Natl. Acad. Sci.* **114**, 11097–11102 (2017).
48. Gavrilov, M. & Bechhoefer, J. Erasure without work in an asymmetric double-well potential. *Phys. Rev. Lett.* **117**, 29–32 (2016).
49. Barker, A. S., Ditzenberger, J. A. & Remeika, J. P. Lattice vibrations and ion transport spectra in β-alumina. II. Microwave spectra. *Phys. Rev. B* **14**, 4254–4265 (1976).
50. Hoppe, R., Kloidt, T. & Funke, K. Frequency-Dependent Conductivities of $RbAg_4I_5$ and Na-β″-Alumina from Radio to FIR Frequencies. *Berichte der Bunsengesellschaft für Phys. Chemie* **95**, 1025–1028 (1991).
51. Kamishima, O., Iwai, Y. & Kawamura, J. Small power-law dependence of ionic conductivity and diffusional dimensionality in β-alumina. *Solid State Ionics* **281**, 89–95 (2015).
52. Suemoto, T. & Ishigame, M. Quasielastic Light Scattering in superionic β-alumina. *Phys. Rev. B* **32**, 4126 (1985).
53. Kawaharada, I., Hattori, T., Ishigame, M. & Shin, S. Quasielastic light scattering in $Na_{1-x}Ag_x$ β-aluminas. *Solid State Ionics* **69**, 79–84 (1994).
54. Kamishima, O. *et al.* Temperature dependence of low-lying phonon dephasing by ultrafast spectroscopy (optical Kerr effect) in Ag β-alumina and Tl β-alumina. *J. Phys. Condens. Matter* **19**, 456215 (2007).
55. Allen, S. J. & Remeika, J. P. Direct measurement of the attempt frequency for ion diffusion in Ag and Na β-alumina. *Phys. Rev. Lett.* **33**, 1478–1481 (1974).
56. Hayes, W. Light scattering by superionic conductors. in *Light Scattering in Solids III. Topics in Applied Physics.* vol. 51 93–120 (Springer Berlin Heidelberg, 1982).
57. McWhan, D. B., Shapiro, S. M., Remeika, J. P. & Shirane, G. Neutron-scattering studies on beta-alumina. *J. Phys. C Solid State Phys.* **8**, L487 (1975).
58. Lucazeau, G., Dohy, D., Fanjat, N. & Dianoux, A. J. Study of the dynamics of a single crystal of Na^+ β-Al_2O_3 by neutron scattering. *Solid State Ionics* **28–30**, 1611–1616 (1988).
59. Bjorkstam, J. L. & Villa, M. NMR studies of superionic β-aluminas. *J. Phys.* **42**, 345–351 (1981).

60. Bjorkstam, J. L., Villa, M. & Farrington, G. C. Temperature dependence of the Na+ distribution in β-aluminas. *Solid State Ionics* **5**, 153–156 (1981).
61. Greenbaum, S. G. & Strom, U. Low-temperature nuclear spin relaxation in β-aluminas. *Solid State Commun.* **46**, 437–440 (1983).
62. Vineyard, G. H. Frequency factors and isotope effects in solid state rate processes. *J. Phys. Chem. Solids* **3**, 121–127 (1957).
63. Kadkhodaei, S. & Davariashtiyani, A. Phonon-assisted diffusion in bcc phase of titanium and zirconium from first principles. *Phys. Rev. Mater.* **4**, 043802 (2020).
64. de Klerk, N. J. J., van der Maas, E. & Wagemaker, M. Analysis of Diffusion in Solid-State Electrolytes through MD Simulations, Improvement of the Li-Ion Conductivity in β-Li_3PS_4 as an Example. *ACS Appl. Energy Mater.* **1**, 3230–3242 (2018).
65. Ohno, S. *et al.* Materials design of ionic conductors for solid state batteries. *Prog. Energy* **2**, 022001 (2020).
66. Muy, S., Schlem, R., Shao-Horn, Y. & Zeier, W. G. Phonon–Ion Interactions: Designing Ion Mobility Based on Lattice Dynamics. *Adv. Energy Mater.* **11**, 2002787 (2021).
67. Krauskopf, T. *et al.* Comparing the Descriptors for Investigating the Influence of Lattice Dynamics on Ionic Transport Using the Superionic Conductor $Na_3PS_{4-x}Se_x$. *J. Am. Chem. Soc.* **140**, 14464–14473 (2018).
68. Muy, S. *et al.* Tuning mobility and stability of lithium ion conductors based on lattice dynamics. *Energy Environ. Sci.* **11**, 850–859 (2018).
69. Morgan, B. J. & Madden, P. A. Relationships between atomic diffusion mechanisms and ensemble transport coefficients in crystalline polymorphs. *Phys. Rev. Lett.* **112**, 4–6 (2014).
70. Shapiro, S. M. & Reidinger, F. Neutron Scattering Studies of Superionic Conductors. in *Physics of Superionic Conductors* (ed. Salamon, M. B.) 45–75 (Springer-Verlag, 1979).
71. Wakamura, K. Roles of phonon amplitude and low-energy optical phonons on superionic conduction. *Phys. Rev. B.* **56**, 11593–11599 (1997).
72. Wakamura, K. Origin of the low-energy mode in superionic conductors. *Phys. Rev. B.* **59**, 3560–3568 (1999).
73. Bachman, J. C. *et al.* Inorganic Solid-State Electrolytes for Lithium Batteries: Mechanisms and Properties Governing Ion Conduction. *Chem. Rev.* **116**, 140–162 (2016).
74. Greenbaum, S. G., Strom, U. & Rubinstein, M. NMR study of low-energy excitations in Na β-alumina. *Phys. Rev. B* **26**, 5226–5229 (1982).
75. Funke, K. Ion dynamics and correlations. *Philos. Mag. A* **68**, 711–724 (1993).
76. Ngai, K. L. & Strom, U. High-frequency dielectric loss of Na beta-alumina: Evidence for relaxation crossover. *Phys. Rev. B* **38**, 10350–10356 (1988).
